# Effect of *Zingiber officinale* on Lipid Profile and Some Inflammatory Markers in Diabetic Hemodialysis Patients: A Randomized Double-Blind Placebo-Controlled Clinical Trial

**DOI:** 10.1155/2023/7154172

**Published:** 2023-05-16

**Authors:** Parisa Veisi, Helya Rostamkhani, Bahram Niknafs, Mohammad Asghari Jafarabadi, Zohreh Ghoreishi

**Affiliations:** ^1^Student Research Committee, Tabriz University of Medical Sciences, Tabriz, Iran; ^2^Department of Clinical Nutrition, Faculty of Nutrition and Food Sciences, Tabriz University of Medical Sciences, Tabriz, Iran; ^3^Kidney Research Center, Tabriz University of Medical Sciences, Tabriz, Iran; ^4^Cabrini Research, Cabrini Health, 154 Wattletree Rd, Melbourne, VIC 3144, Australia; ^5^School of Public Health and Preventative Medicine, Faculty of Medicine, Nursing and Health Sciences, Monash University, Melbourne, VIC, 3004, Australia; ^6^Department of Psychiatry, School of Clinical Sciences, Faculty of Medicine, Nursing and Health Sciences, Monash University, Clayton, VIC, 3168, Australia; ^7^Road Traffic Injury Research Center, Tabriz University of Medical Sciences, Tabriz, Iran; ^8^Nutrition Research Center, Department of Clinical Nutrition, Faculty of Nutrition and Food Sciences, Tabriz University of Medical Sciences, Tabriz, Iran

## Abstract

**Background:**

Diabetes, inflammation, and abnormal lipid levels are the main risk factors for mortality in end-stage renal disease (ESRD). The present study aimed to investigate the effects of ginger supplementation on inflammatory markers and lipid profile in diabetic patients with ESRD undergoing hemodialysis.

**Methods:**

In this study, 44 patients were randomly assigned to either the ginger or the placebo group. The patients in the ginger group received 2000 mg/d ginger for eight weeks, while the control group received the placebo with the same protocol. The serum concentrations of triglyceride (TG), total cholesterol (TC), low-density lipoprotein-cholesterol (LDL-c), high-density lipoprotein-cholesterol (HDL-c), albumin, and high-sensitivity C-reactive protein (hs-CRP) were measured after a 12- to 14-hours fast at the baseline and the end of the study, as along with the platelet-to-lymphocyte ratio (PLR), neutrophil-to-lymphocyte ratio (NLR), and Glasgow prognostic score (GPS).

**Results:**

Forty-one subjects were analyzed based on the intention-to-treat method of all included patients. Serum levels of TG (*p*=0.003), hs-CRP (*p*=0.022), and NLR (*p*=0.001) decreased significantly in the ginger group compared to the placebo group, while albumin concentration in serum was elevated (*p*=0.022). However, there were no significant differences in GPS, levels of TC, LDL-C, HDL-C, and PLR within and between the groups (*p* > 0.05).

**Conclusion:**

Ginger administration reduced NLR, hs-CRP, and TG serum levels and increased serum albumin levels in included patients. Thus, ginger can be considered an effective complementary treatment for these patients. This trail is registered with IRCT20191109045382N3.

## 1. Introduction

End-stage renal disease (ESRD) is defined as having a glomerular filtration rate of less than 15 mL/min [1]. Among all influencing parameters that elevate ESRD risk, diabetes mellitus has a more pronounced impact [2, 3].

Diabetic ESRD patients challenge nephrologists as they have a significant number of comorbidities [3, 4]. Cardiovascular diseases are the leading cause of increased mortality in these patients [4]. General risk factors such as hypertension and lipid profile disorders alone cannot justify the high prevalence of cardiovascular disease in these patients, so other risk factors such as oxidative stress, inflammation, and insulin resistance should be considered, though they may cause development or aggravation of cardiovascular disease as well [5, 6].

Systemic inflammation plays a fundamental role in kidney damage; thus, finding a method to attenuate inflammation in ESRD diabetic patients seems necessary [7]. Tsirpanlis reported that serum levels of high-sensitivity C-reactive protein (hs-CRP) in ESRD patients could estimate their overall health status [8]. Elevated serum CRP and interleukin-6 (IL-6) levels have been reported to positively affect mortality in this group of patients [9–11]. It has also been shown that the neutrophil-to-lymphocyte ratio (NLR), as a marker of subclinical inflammation, is associated with endothelial dysfunction in patients with chronic kidney disease, and it rises in patients who are on predialysis or dialysis procedures [12]. There is also a direct link between NLR, hs-CRP, and IL-6, which can indicate inflammation in ESRD patients [13]. Another piece of evidence shows that the platelet-to-lymphocyte ratio (PLR), which has been used in many diseases for predicting inflammation and mortality, has emerged as an informative marker revealing changes in platelet and lymphocyte counts due to acute inflammatory and prothrombotic states in kidney diseases [14]. In addition, many studies have suggested that the Glasgow prognostic score (GPS) is a good predictor of poor survival in kidney disease patients and predicts treatment outcomes [15]. GPS is computed from the combination of CRP and serum levels of albumin, which predicts poor prognoses in many diseases, such as cancer and coronary artery disease [16].

In recent years, the use of complementary and alternative medicine for the management of chronic diseases has become popular [17–19]. Also, nutrition therapy and adjunct medication strategies have proven to be reliable methods for controlling the disease in diabetic patients with ESRD [7, 20]. Regarding their anti-inflammatory effects, spices are of great importance. Ginger (*Zingiber officinale* Roscoe) is a nontoxic spice that has been extensively used in traditional Chinese, Indian, Persian, and Greek medicine [21, 22]. It seems that the beneficial effects of ginger relate to its potential antioxidant, anticarcinogenic, and anti-inflammatory properties [21, 22], which could be of utmost importance in controlling diabetes and kidney function [22, 23]. Some studies have also demonstrated the beneficial effects of ginger on the lipid profile [24–26]. At the same time, the results are controversial regarding the hyperinflammatory status and dyslipidemia in diabetic patients undergoing hemodialysis, which elevate the risk of mortality. Given the potential anti-inflammatory properties of ginger, this study aimed to assess this herb's effect on inflammatory markers, lipid profiles, and nutritional status in this group of patients.

## 2. Materials and Methods

### 2.1. Study Setting

This randomized, double-blinded, placebo-controlled study was conducted in the Dialysis Center of the Imam Reza Hospital affiliated with Tabriz University of Medical Sciences, East Azerbaijan, Tabriz, Iran. The Research Ethics Committees of Tabriz University of Medical Sciences approved the trial protocol (IR.TBZMED.REC.1398.1186), and it was registered in the Iranian Registry of Clinical Trials (IRCT), which is a primary registry in the WHO Registry Network (IRCT20191109045382N3).

### 2.2. Study Population

**Figure 1:**
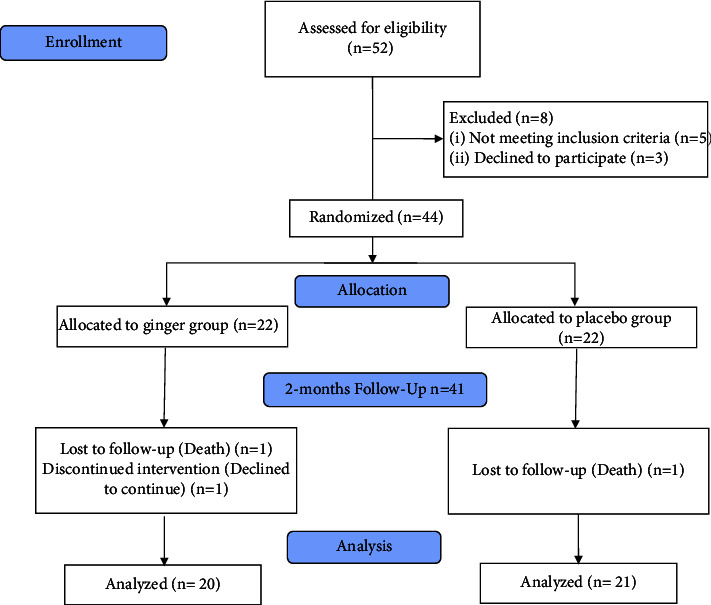
CONSORT flow diagram of the study.

Patients were enrolled in this study if they were 18 years or older, diagnosed with T2DM, and needed hemodialysis for at least the last three months based on a 2- or 3-times weekly schedule (each series for 4 hours). However, the following conditions constituted the exclusion criteria: acute gastrointestinal diseases; thyroid disorders; gallstones; a history of allergy to ginger; those who were taking fish oil supplements, steroidal and nonsteroidal anti-inflammatory drugs, levothyroxine, warfarin, antioxidant supplements, and ginger in the form of pills or capsules one month before the initiation of the study. Taking ginger as a spice added to foods in minimal amounts was considered the exception. Any changes in the dialysis program during the study (changing hemodialysis to peritoneal dialysis or kidney transplantation), irregular attendance in hemodialysis sessions, and unwillingness to continue the study were other exclusion criteria.  Figure 1 shows the study flow diagram based on the CONSORT.

### 2.3. Study Protocol

A computer-generated randomization procedure was used for assigning patients to one of the two arms of the study. The patients were appropriately matched based on age (age < 50, 50 ≤ age) and gender (male, female) and blood sugar (FBS < 200, 200 ≤ FBS) to minimize cofounders. Permuted block randomization was used to allocate the patients in the study arms. The participants in the ginger group took 2000 mg of ginger powder (Goldaroo, Co. Isfahan, Iran) in four 500 mg capsules daily for eight weeks, while patients in the control group received four identical capsules containing starch. Each patient was provided with a sufficient number of capsules for weekly consumption, and they were advised to return the unused capsules each week to ascertain patient compliance. To keep the study double-blind, neither the field executive researcher nor the study participants were aware of the patient's assignment to the study arms. Both groups of patients continued regular hemodialysis, two or three sessions a week.

### 2.4. Biochemical Analyses

Blood samples (7 ml) were taken prior to hemodialysis after a 12 h to 1 kh fasting and kept at room temperature (20°C–25 C) for 20 min. After clotting, the samples were centrifuged at 2000*g* for 10 min; separated serum samples were divided into small aliquots and were frozen at −70°C for future biochemical analyses done at the baseline and after eight weeks. Lipid profiles, including total cholesterol (TC), triglyceride (TG), and high-density lipoproteins (HDL-C), as well as serum albumin, were measured by an enzymatic spectrophotometric method using the Autoanalyzer (Alcyon 300, Abbott Park, Illinois) with Pars-Azmoon kit (Tehran, Iran). Serum low-density lipoprotein (LDL-C) was estimated using the Friedwald equation [27]. Additionally, the hs-CRP level was determined by a Pars-Azmoon kit (Tehran, Iran) and the Alcyon 300 automated analyzer kit based on the turbidimetric method. Complete blood count (CBC) with differential was also measured using the Sysmex XP 300N automated hematology analyzer (Sysmex, Kobe, Japan) for calculating NLR and PLR. Another score used for inflammatory analysis was the GPS that was calculated by giving one point to elevated hs-CRP (>10 mg/L) and lowered albumin (<3.5 mg/dl). If patients score zero on all items, their final GPS score would be zero, and if one or both parameters were abnormal, they would score 1 or 2, respectively, with a score of 2 indicating the highest degree of inflammation [28].

### 2.5. Assessment of Other Variables

General characteristics of the patients and anthropometric indices of weight, height, body mass index (BMI), waist circumference, and hip circumference were measured using standard methods. A short version of the International Physical Activity Questionnaire (IPAQ) was used to assess physical activity levels, whose validity and reliability have been reported previously [29]. The IPAQ was scored based on the recognized methods [30], and data were reported as a metabolic equivalent of task minutes per week [14]. Additionally, the dietary intakes of the patients were evaluated using a 3-day dietary recall (2 days during the week and one day on the weekend) after providing them with a full description, necessary education, and practical samples. The obtained results of diet were analyzed by Nutritionist IV software (N-Squared Computing, San Bruno, CA, USA). All measurements were carried out at the baseline and after eight weeks.

### 2.6. Sample Size

The minimum sample size was calculated as ten based on data acquired from the previous study [31] with a confidence interval of 95 percent, power of 95%, and *α* = 0.05, and total cholesterol as a key variable using G^*∗*^Power software version 3.1.9.6. Finally, a dropout rate of 30% was considered, which increased the sample size to 15 per group.

### 2.7. Statistical Analyses

Statistical measurements were performed using SPSS version 21.0 (SPSS Inc., Chicago, IL, USA) based on intention-to-treat analysis, with the normality distributions of variables evaluated using the Kolmogorov–Smirnov test. The baseline comparison was made by the Student's *t*-test and Mann–Whitney *U* test for quantitative variables with or without normal distribution. In contrast, the chi-square test was used for qualitative variables. Analysis of covariance (ANCOVA), adjusted for confounding variables, was performed to assess between-group differences. The paired samples *t*-test and Wilcoxon signed-rank test were used to investigate within-group changes if the variables had a normal distribution and not, respectively. A *p* value less than or equal to 0.05 was considered significant.

## 3. Results

A total of 44 participants were randomly assigned to either the ginger (*n* = 22) or placebo (*n* = 22) study groups. However, forty-one participants completed the study and were included in the statistical analysis based on the intention-to-treat method. After visits, it was noted that a slightly variable number of capsules were returned; however, the compliance degree was acceptable, and no adverse events were reported.

The general characteristics of the study participants have been presented in Table 1. There were no statistically significant differences between the two groups regarding demographic, anthropometric, and drug histories before the study's initiation (*p* > 0.05). No significant between-group differences were observed either for energy or macronutrient intake (*p* > 0.05; Table 2), and so were other variables of weight, BMI, waist circumference, hip circumference, and physical activity levels before and after the intervention (*p* > 0.05; Table 3).

Analysis of covariance revealed that the reduction of TG (14%) in the ginger group was statistically significant compared to the placebo group adjusted for baseline measurements of calorie intake, weight change, and antilipid drugs (*F* (1, 35) = 8.839, *p* = 0.005). Furthermore, it was significantly reduced only in the ginger group at the end of the study based on a within-group assessment (*p* = 0.003). Notably, ginger supplementation could reduce serum concentration of TC by 8% and LDL-C by 12% and increase HDL-C levels by 1%, though these changes did not reach statistical significance (Table 4).

The difference between the serum levels of hs-CRP in the ginger and placebo groups was significant (*F* (1, 36) = 5.392, *p*=0.026 after adjusting for confounders), while it was significantly reduced in the ginger group (*p*=0.022) at the end of the study, with no significant changes observed in the placebo group. Similarly, serum albumin concentrations were elevated significantly in the ginger group (*p*=0.022), and the between-group differences were remarkable as well (*F* (1, 36) = 8.614, *p*=0.006, adjusting for confounders) (Table 4).

**Figure 2:**
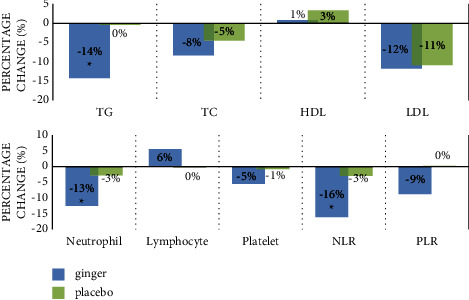
Change percent of the measured parameters within the study groups. TG = triglycerides; TC = total cholesterol; HDL = high-density lipoprotein; LDL = low-density lipoprotein; NLR = neutrophil lymphocyte ratio; PLR = platelet lymphocyte ratio.

Furthermore, the NLR dropped significantly within the ginger group (*p*=0.001), and there was a significant difference between the two study groups (*F* (1, 36) = 4.376, *p*=0.044). However, the PLR did not change remarkably within and between groups (*p* > 0.05; Table 4). No significant difference was found either in the GPS score before and after the intervention (*p* > 0.05; Table 5).  Figure 2 illustrates the percentage change in the metabolic profile of study participants in the ginger and placebo groups.

## 4. Discussion

This study examined the effect of short-term supplementation with ginger on the lipid profile and inflammatory status of diabetic patients with ESRD undergoing hemodialysis. It was revealed that ginger supplementation caused a 14% reduction in serum levels of TG with no significant effects on HDL-C, LDL-C, and TC. This result is consistent with Tabibi et al.'s study in 2015, which investigated the effect of taking 1000 mg of ginger for ten weeks in patients undergoing peritoneal dialysis [31]. In Arablou et al.'s study in 2014, ginger reduced TG and TC in diabetic patients, but it had no significant effect on HDL-C and LDL-C levels [24]. In another study accomplished by Carvalho et al., daily administration of 1.2 g of ginger for 90 days did not affect serum TG and HDL-C concentrations but significantly lowered TC and LDL-C in patients with T2DM [32]. Furthermore, a meta-analysis by Jafarnejad et al. showed that ginger intake significantly reduced the concentrations of TG and TC and significantly elevated HDL-C in diabetic and hyperlipidemic subjects [33].

Several animal studies have confirmed the hypolipidemic effects of ginger [26, 34, 35]. A possible mechanism of the hypotriglyceridemic effects of ginger may be due to enhancing lipoprotein lipase activity, resulting in the hydrolysis of circulatory TG and decreasing serum TG [36]. Moreover, one of the nutrients in ginger is niacin, which may have the ability to reduce serum triglyceride levels [37]. In contrast to the majority of previous studies, the current study results showed an insignificant lowering effect of ginger on TC and LDL-C [24, 32, 38, 39]. This inconsistency may be due to administering different doses of ginger or different intervention durations. In addition, in this study, about 30% of patients had received antilipid medications such as atorvastatin to control cholesterol levels.

Low-grade inflammation is a common feature of diabetes, which plays a major role in its prognosis and the pathogenesis of secondary complications such as nephropathy. To our knowledge, no research has investigated the effect of ginger on serum hs-CRP in diabetic hemodialysis patients. However, Imani et al. reported that daily supplementation with 1000 mg of ginger for ten weeks had no significant effect on serum hs-CRP in peritoneal dialysis patients [40]. On the contrary, most evidence indicates that ginger administration significantly lowers serum levels of hs-CRP [24, 41, 42], which agrees with our result. Ginger reduces inflammation through NF-*κ*B signaling pathway suppression and inhibition of prostaglandin synthesis [43].

In our study, daily administration of 2000 mg ginger significantly elevated serum albumin (by 8%) in patients with diabetes and ESRD. To date, no studies have examined the effects of ginger consumption on serum albumin concentrations in this group of patients. However, current results are in line with previous animal studies indicating the enhancing effects of ginger on serum levels of albumin in acute kidney injury rats [44, 45]. In a study by Al Shammari on male rats with induced kidney damage, ginger supplementation significantly elevated serum albumin and protein [45]. In disagreement with our result showing increased serum albumin, the Seddik study in 2015 showed that daily supplementation of 1500 mg of ginger for eight weeks in hemodialysis patients did not significantly change serum aluminum levels [46]. These pieces of evidence show that ginger consumption may increase the protein synthesis rate in the liver and decrease albuminuria, leading to a higher serum concentration of albumin. However, the GPS score of the patients in the ginger group was not different from that of the placebo group.

Previous studies have shown that NLR and PLR positively correlate with inflammation markers such as hs-CRP, IL-6, and tumor necrosis factor-alpha (TNF-*α*) [14, 47, 48]. At the same time, PLR is better than NLR for predicting inflammation [14]. In addition, Turkmen et al. found a negative correlation between NLR and serum albumin, as well as TC levels [48]. The present data show that ginger supplementation for eight weeks can significantly reduce the NLR (by 16%) compared with the placebo group, but the reduction of the PLR (9%) was insignificant. While there are limited data about the effect of ginger on NLR and PLR, Ali et al. stated that 6-gingerol, an active constituent of ginger, elevates intracellular concentrations of cAMP and enhances protein kinase A activity in neutrophils by suppressing reactive oxygen species formation through a mechanism partially dependent on inhibition of phosphodiesterase activity, which attenuates neutrophil hyperactivity [49].

Studies on hemodialysis patients have shown that survival increases and decreases with increasing BMI and WC, respectively [50–52]. Azimi et al. reported that daily supplementation with 3000 mg of ginger for eight weeks had no significant effect on BMI and WC in T2DM patients [53]. Also, Tabibi et al. reported that daily supplementation with 1000 mg of ginger for ten weeks had no significant effect on BMI in peritoneal dialysis patients, which agrees with our result [31].

To the best of our knowledge, this is the first study investigating the effects of ginger supplementation on diabetic hemodialysis patients. The nonmeasurement of proinflammatory cytokines, including IL-1*β*, IL-6, and TNF-*α*, due to financial constraints was one of the study's limitations. Also, hemodialysis patients' adherence to ginger supplementation may have been poor due to old age and memory impairment. We visited them weekly, and we tried to resolve this problem. Notably, the short duration of supplementation (eight weeks) was another limitation of this study and the reason for some of the insignificant changes at the end of the research project.

## 5. Conclusion

This study revealed that supplementation with ginger for eight weeks in diabetic hemodialysis patients had beneficial effects on serum TG levels, albumin, and overall inflammatory status. Further clinical studies comparing different dosages, various forms of ginger, and a longer duration of supplementation are warranted.

## Figures and Tables

**Table 1:** Baseline characteristics of the patients in intervention and control groups.

Variables	Ginger group (*n* = 22)	Placebo group (*n* = 22)	*p* value
Age (years)	60.05 ± 11.12	59.64 ± 10.69	0.902^*∗*^
Sex			0.763^†^
Men (%)	11 (50%)	12 (54.5%)	
Women (%)	11 (50%)	10 (45.5%)	
Height [28]	161.46 ± 8.70	161.76 ± 9.62	0.916^*∗*^
Weight [12]	69.67 ± 10.76	74.55 ± 14.31	0.209^*∗*^
BMI (kg·m^−2^)	26.48 ± 3.71	28.41 ± 4.35	0.123^*∗*^
Dialysis frequency			0.709^†^
Two sessions per week	4 (18.2%)	5 (22.7%)	
Three sessions per week	18 (81.8%)	17 (77.3%)	
Drug history			
Antilipids	6 (27.3%)	7 (31.8%)	0.741^†^

Values are reported as mean ± SD or median (IQR) for quantitative data and frequency (percentage) for qualitative data. ^*∗*^Independent *T*-test, ^#^Mann–Whitney U test, ^†^chi-square test.

**Table 2:** Comparison of mean energy intake and macronutrients in the study patients before and after intervention.

Variables	Ginger group (*n* = 20)	Placebo group (*n* = 21)	Mean difference (CI 95%)	*p* value
Energy (Kcal/d)				
Baseline	1628.95 ± 292.04	1646.77 ± 242.23	−17.82 (−181.23, 145.60)	0.827^†^
Endpoint	1650.55 ± 276.88	1644.02 ± 232.52	10.12 (−55.69, 75.94)	0.757^††^
Mean difference (CI 95%)	24.00 (−36.24, 84.24)	13.28 (−26.82, 53.38)		
*p* value^*∗*^	0.415	0.498		
Carbohydrates (g/d)				
Baseline	216.77 ± 41.20	219.30 ± 37.88	−2.52 (−26.60, 21.56)	0.834^†^
Endpoint	220.75 ± 39.04	219.92 ± 36.32	2.91 (−11.10, 16.92)	0.676^††^
Mean difference (CI 95%)	3.97 (−6.74, 14.69)	0.22 (−9.93, 10.36)		
*p* value^*∗*^	0.449	0.965		
Protein (g/d)				
Baseline	52.42 ± 11.11	54.73 ± 9.11	−2.31 (−8.66, 4.04)	0.467^†^
Endpoint	52.78 ± 10.23	54.18 ± 7.65	−0.66 (−4.79, 3.47)	0.748^††^
Mean difference (CI 95%)	0.25 (−3.21, 3.71)	0.44 (−3.07, 3.95)		
*p* value^*∗*^	0.881	0.796		
Fat (g/d)				
Baseline	63.17 ± 12.17	63.07 ± 12.26	0.09 (−6.78, 6.94)	0.979^†^
Endpoint	64.41 ± 12.13	63.94 ± 10.49	0.15 (−4.02, 4.33)	0.941^††^
Mean difference (CI 95%)	1.18 (−1.88, 4.24)	1.11 (−2.21, 4.43)		
*p* value^*∗*^	0.429	0.495		

Values are reported as mean ± SD. ^*∗*^Paired samples *t*-test, ^†^independent samples *T*-test, ^††^ANCOVA adjusted for baseline values.

**Table 3:** Comparison of anthropometric indices in the study patients before and after intervention.

Variables	Ginger group (*n* = 20)	Placebo group (*n* = 21)	Mean difference (CI 95%)	*p* value
Weight [12]				
Baseline	69.67 ± 10.76	74.55 ± 14.31	−4.88 (−12.58, 2.85)	0.209^†^
Endpoint	69.79 ± 10.38	74.36 ± 15.21	−0.02 (−0.92, 0.88)	0.965^††^
Mean difference (CI 95%)	−0.28 (−0.93, 0.36)	−0.12 (−0.77, 0.54)		
*p* value^*∗*^	0.370	0.704		
BMI (kg/m^2^)				
Baseline	26.48 ± 3.71	28.41 ± 4.35	−1.92 (−4.38, 0.54)	0.123^†^
Endpoint	26.50 ± 3.90	28.40 ± 4.58	−0.14 (−0.36, 0.33)	0.934^††^
Mean difference (CI 95%)	−0.11 (−0.36, 0.14)	−0.06 (−0.30, 0.18)		
*p* value^*∗*^	0.364	0.614		
Waist circumference [28]				
Baseline	99.08 ± 10.14	104.18 ± 10.44	0.53 (−4.71, 3.63)	0.108^†^
Endpoint	99.05 ± 9.34	104.07 ± 11.3	0.09 (−0.57, 0.75)	0.780^††^
Mean difference (CI 95%)	−0.34 (−0.80, 0.11)	−0.31 (−0.78, 0.17)		
*p* value^*∗*^	0.133	0.189		
Hip circumference [28]				
Baseline	98.88 ± 8.05	101.92 ± 9.48	−3.04 (−8.39, 2.32)	0.258^†^
Endpoint	98.73 ± 8.01	101.99 ± 9.88	0.06 (−0.28, 0.40)	0.541^††^
Mean difference (CI 95%)	−0.14 (−0.36, 0.07)	−0.21 (−0.51, 0.09)		
*p* value^*∗*^	0.182	0.167		
Waist to hip ratio				
Baseline	1.02 (0.94, 1.05)	1.03 (1.01, 1.05)	—	0.346^#^
Endpoint	1.02 (0.95, 1.04)	1.02 (1.01, 1.05)	—	0.401^#^
Mean difference (CI 95%)	—	—		
*p* value^*∗∗*^	0.477	0.404		
Physical activity (MET-min/week)				
Baseline	310.50 (143.75, 594.00)	241 (198.75, 297.00)	—	0.533^#^
Endpoint	355.50 (161.25, 583.00)	266 (198.50, 323.00)	—	0.308^#^
Mean difference (CI 95%)	—	—		
*p* value^*∗∗*^	0.091	0.249		

Values are reported as mean ± SD or median (IQR) for quantitative data. ^*∗*^Paired samples *t*-test, ^†^independent samples *t*-test, ^#^Mann–Whitney U test, ^*∗∗*^Wilcoxon signed-rank test, ^††^ANCOVA adjusted for baseline values and energy intake. BMI: body mass index.

**Table 4:** Overall metabolic parameters of study patients before and after intervention.

Variables	Ginger group (*n* = 20)	Placebo group (*n* = 21)	Mean difference (CI 95%)	*p* value
Triglyceride (mg/dL)				
Baseline	104.27 ± 22.65	113.59 ± 36.50	−9.32 (−27.80, 9.16)	0.315†
Endpoint	89.45 ± 24.28	113.14 ± 30.10	−16.59 (−27.92, 5.26)	**0.005** ^**†††**^
Mean difference (CI 95%)	−14.20 (−23.03, −5.37)	−0.67 (−10.83, 9.50)		
*p* value^*∗*^	**0.003**	0.893		
Total cholesterol (mg/dL)				
Baseline	129.05 ± 36.01	129.41 ± 38.85	−0.36 (−23.15, 22.43)	0.974^†^
Endpoint	118.35 ± 30.50	123.57 ± 33.36	−5.27 (−20.50, 9.97)	0.488^†††^
Mean difference (CI 95%)	−5.65 (−13.77, 2.47)	−3.67 (−18.91, 11.58)		
*p* value^*∗*^	0.162	0.621		
HDL-C (mg/dL)				
Baseline	39.64 ± 8.63	41.00 ± 11.39	1.36 (−7.51, 4.78)	0.657^†^
Endpoint	39.95 ± 9.99	42.38 ± 10.70	−0.24 (−4.56, 4.08)	0.910^†††^
Mean difference (CI 95%)	1.40 (−1.29, 4.09)	1.38 (−2.06, 4.83)		
*p* value^*∗*^	0.289	0.413		
LDL-C (mg/dL)				
Baseline	68.55 ± 31.84	65.69 ± 31.90	2.86 (−16.53, 22.26)	0.767^†^
Endpoint	60.51 ± 27.05	58.56 ± 28.28	−1.48 (−14.51, 11.56)	0.819^†††^
Mean difference (CI 95%)	−4.21 (−11.95, 3.53)	−4.91 (−17.82, 7.80)		
*p* value^*∗*^	0.269	0.436		
hs-CRP (mg/L)				
Baseline	8.06 ± 3.49	6.32 ± 3.68	1.74 (−0.44, 3.92)	0.115^†^
Endpoint	6.57 ± 3.79	7.35 ± 3.67	−1.87 (−3.50, −0.24)	**0.026** ^**††**^
Mean difference (CI 95%)	−1.58 (−2.89, −0.26)	0.92 (−0.09, 1.93)		
*p* value^*∗*^	**0.022**	0.072		
Albumin (g/dl)				
Baseline	4.32 ± 0.50	4.16 ± 0.87	0.16 (−0.26, 0.58)	0.455^†^
Endpoint	4.66 ± 0.52	4.04 ± 0.71	0.59 (0.18, 0.99)	**0.006** ^**††**^
Mean difference (CI 95%)	0.35 (0.06, 0.63)	−0.13 (−0.57, 0.32)		
*p* value^*∗*^	**0.022**	0.558		
NLR				
Baseline	2.01 ± 0.58	2.08 ± 0.52	−0.07 (−0.41, 0.26)	0.657^†^
Endpoint	1.68 ± 0.50	2.02 ± 0.54	−0.26 (−0.51, −0.01)	**0.044** ^**††**^
Mean difference (CI 95%)	−0.28 (−0.43, −0.14)	−0.01 (−0.24, 0.22)		
*p* value^*∗*^	**0.001**	0.925		
PLR				
Baseline	112.41 ± 43.23	112.66 ± 30.98	−0.25 (−23.13, 22.63)	0.982^†^
Endpoint	102.51 ± 41.23	112.88 ± 34.81	−7.56 (−19.32, 4.20)	0.200^††^
Mean difference (CI 95%)	−6.91 (−14.26, 0.44)	1.84 (−7.48, 11.16)		
*p* value^*∗*^	0.064	0.685		

Values are reported as mean ± SD or median (IQR) for quantitative data. ^*∗*^Paired samples *t*-test;^†^independent samples *t*-test;^#^Mann–Whitney U test; ^*∗∗*^Wilcoxon signed-rank test; ^††^ANCOVA adjusted for baseline values, calorie intake, and ∆weight; ^†††^ANCOVA adjusted for baseline values, antilipid drug intake, calorie intake, and ∆weight. NLR: neutrophil-to-lymphocyte ratio; PLR: platelet-to-lymphocyte ratio.

**Table 5:** Glasgow prognostic scores (GPS) of study patients before and after intervention.

Variables	Ginger group (*n* = 20)			Placebo group (*n* = 21)			*p* value^†^
GPS	0	1	2	0	1	2	
Baseline *n* (%)	14 (63.6%)	8 (36.4%)	0 (0%)	17 (77.3%)	4 (18.2%)	1 (4.5%)	0.269
Endpoint *n* (%)	16 (80%)	4 (20%)	0 (0%)	12 (57.1%)	7 (33.3%)	2 (9.5%)	0.186
*p* value^*∗∗*^	0.157			0.160			

^†^chi-square test, ^*∗∗*^Wilcoxon signed-rank test.

## Data Availability

The datasets used and analyzed during the current study are available from the corresponding author upon reasonable request.
